# Is Adult Second Language Acquisition Defective?

**DOI:** 10.3389/fpsyg.2020.01839

**Published:** 2020-07-30

**Authors:** Ewa Dąbrowska, Laura Becker, Luca Miorelli

**Affiliations:** ^1^Chair of Language and Cognition, Department of English and American Studies, Friedrich-Alexander-Universität Erlangen-Nürnberg, Erlangen, Germany; ^2^Department of English Language and Linguistics, University of Birmingham, Birmingham, United Kingdom

**Keywords:** second language acquisition, ultimate attainment, age effects, fundamental difference hypothesis, individual differences, grammaticality judgment task, picture selection task, “decorative” grammar

## Abstract

There is a large literature showing that adult L2 learners, in contrast to children, often fail to acquire native-like competence in the second language. Because of such age effects, adult L2 learning is often viewed as “fundamentally different” from child acquisition and defective in some way. However, adult L2 learners do not always do worse than child learners. Several studies (e.g., Sasaki, 1997; Dąbrowska and Street, 2006; Street, 2017; Dąbrowska, 2019) found considerable overlap between L1 and L2 speakers’ performance on tasks tapping morphosyntactic knowledge. Crucially, these studies used grammatical comprehension tasks (e.g., picture selection) to test mastery of “functional” grammar (i.e., grammatical contrasts which correspond to a clear difference in meaning, such as the assignment of agent and patient roles in sentences with noncanonical word order and quantifier scope). In contrast, most ultimate attainment studies (e.g., Johnson and Newport, 1989; Flege et al., 1999; DeKeyser, 2000; DeKeyser et al., 2010) used a grammaticality judgment task (GST) which assessed mastery of “decorative” grammar, i.e., grammatical morphemes such as tense and agreement markers which make relatively little contribution to the meaning conveyed by a sentence. In this study, we directly compared native speakers, late immersion learners, and classroom foreign language learners on tasks assessing both aspects of grammar. As in earlier studies, we found significant differences between native speakers and both non-native groups in performance on “decorative” grammar, particularly when performance was assessed using spoken rather than written stimuli. However, the differences in performance on the “functional” grammar task were much smaller and statistically non-significant. Furthermore, even in the “decorative” grammar task, there was more overlap between native speakers and late L2 learners than reported in earlier research. We argue that this is because earlier studies underestimated the amount of variation found in native speakers.

## Introduction

A large number of studies in the L2 acquisition literature have demonstrated that adult learners often fail to acquire native-like competence in the second language (e.g., Coppieters, 1987; Johnson and Newport, 1989; Johnson et al., 1996; DeKeyser, 2000; Abrahamsson and Hyltenstam, 2009; DeKeyser et al., 2010; Granena and Long, 2013; Han, 2013). Because of these findings, adult second language learning is commonly viewed as “fundamentally different” from child acquisition and defective in some way (see, for example Bley-Vroman, 1989, 2009; DeKeyser, 2000; Han and Selinker, 2005; Han, 2013). The failure of many adult L2 learners to acquire native-like linguistic representations is often attributed to maturational changes in the brain, such as lack of or incomplete access to UG (Bley-Vroman, 1989; Schachter, 1996), or less effective procedural learning (DeKeyser, 2000; Paradis, 2004; Granena and Long, 2013; Ullman, 2015). However, alternative explanations, which appeal to the quantity and quality of input available to the learner (Flege et al., 1999; Flege, 2019) and identification with the L2 community (Schumann, 1986; Preston, 1989) have also been put forward.

Nonetheless, not all studies found the outcome of adult L2 learning to be defective. Dąbrowska and Street (2006), for example, tested the comprehension of plausible actives (*The dog bit the man*), implausible actives (*The man bit the dog*), and plausible and implausible passives by four groups: high academic attainment (HAA) natives, low academic attainment (LAA) natives, HAA non-natives, and LAA non-natives. The task was to identify the “do-er” (i.e., the agent) in each sentence. The authors found that both HAA groups and the less educated non-native group were at ceiling in all conditions. The LAA natives were also at ceiling on the plausible sentences, but only 65% correct on implausible actives and 36% correct on implausible passives. In other words, the LAA native group sometimes resorted to a pragmatic rather than syntactic strategy when confronted with implausible sentences (which could be due to misunderstanding the instructions); however, over and above this, they appeared to have difficulty understanding passive sentences, and as a result, performed less well than both groups of non-native speakers.

Sasaki (1997) used a similar method to test comprehension of active and causative sentences with either canonical or noncanonical word order in Japanese. The non-native participants were L1 English students attending a fourth semester course who had received about 280 h of formal instruction in Japanese; the native participants were likewise university students. On the initial assessment, the native speakers did slightly better overall (70% correct, compared to a mean score of 64% in the non-native group). In the second phase of the experiment, participants were given a training session, which was followed by a posttest. Both groups improved as a result of training, but non-native speakers improved considerably more, so they actually did slightly better on the posttest (87% correct, compared to 81% in the native group).

A more recent study by Street (2017) tested comprehension of simple transitives, subject relatives, and object relatives in English using a picture selection task (PST). Street tested three groups: HAA native speakers, LAA natives, and HAA non-natives from a variety of language backgrounds. All participants were at ceiling on simple transitives and subject relatives. In the object relative condition, the HAA native group performed significantly better than the HAA non-natives (95 vs. 88% correct), who in turn were significantly better than the low LAA natives (71% correct). Importantly, performance in the latter group was very varied, with individual scores ranging from 15 to 100%.

Finally, Dąbrowska (2019) tested comprehension of a variety of constructions (see below) as well as knowledge of vocabulary and collocations. The L1 participants were 90 speakers of varying educational background (from no formal qualifications to PhD, in proportions roughly reflecting the demographics of the UK population). The non-native participants were immersion learners of various language backgrounds who had lived in the UK for at least 3 years (mean 7) with an age of arrival of 16 or above (mean 25). Like the native speakers, they came from a variety of educational backgrounds. As a group, the native speakers performed better than the non-natives; interestingly, the difference was largest for collocations and smallest for grammar. Crucially, however, there was considerable overlap between the L1 and L2 speakers in performance on all three tasks, with 75% of adult L2 learners scoring within the native speaker range on the grammatical comprehension task.

Why should the two sets of studies produce such different results? The four studies which found a large amount of overlap between native and non-native speakers tapped mastery of what we might call “functional” grammar – that is to say, grammatical contrasts which correspond to a difference in meaning, for example, the assignment of agent and patient roles in sentences with noncanonical word order, such as passives, object clefts and object relatives, and quantifier scope (e.g., the contrast between *Every dog is in a basket* and *Every basket has a dog in it*). Related to this, they assessed grammatical knowledge using a comprehension task. In contrast, the studies cited earlier, which found large differences between native and non-native speakers, and little or no overlap between the two groups, used a grammaticality judgment task (GJT) to test knowledge of what we might call “decorative” grammar, that is to say, grammatical structures with relatively low functional load and subcategorization requirements of individual lexical items. Consider, for example, the sentences in (1–4) below from the stimuli used by Johnson and Newport (1989).

1. *John’s dog always wait for him at the corner*.
2. *Last night the old lady die in her sleep*.
3. *Tom is reading book in the bathtub*.
4. *The boys laughed the clown*.

The first three sentences are ungrammatical because an obligatory grammatical morpheme (the third person inflection, the past tense inflection, and the indefinite determiner) is missing. Importantly, the formal exponents of these morphemes (word-final obstruents in the first two cases and a schwa vowel in the third case) have low perceptual salience, and the meanings that they express are abstract and largely redundant, in the sense that they are (in these sentences at least) easily recoverable from context. The fourth example is slightly different, in that the error involves the subcategorizations restrictions of a particular verb (*laugh* is intransitive, yet it is followed by a direct object). The sentence could be made grammatical by adding the preposition *at* which, while it has somewhat more phonetic substance than the missing morphemes in the preceding examples, is also relatively low in salience. All these factors (low salience, bleached meaning, and redundancy) have been shown to make grammatical morphemes more difficult to learn (Goldschneider and DeKeyser, 2001).

It should be stressed that the “functional” vs. “decorative” distinction is a matter of degree. Grammatical morphemes such as past tense markers or determiners clearly do carry meaning. However, the meanings are quite abstract and usually predictable from other parts of the sentence. Furthermore, many languages do not mark distinctions such as tense and definiteness grammatically, and yet this rarely leads to communicative problems.

A number of researchers have suggested that L2 learners focus more on processing meaning than on the details of form. For example, VanPatten suggested the Primacy of Meaning Principle: “Learners process input for meaning before they process it for form” (see VanPatten, 1996, 2004, p. 7). Thus, learners focus more on content words than on grammatical morphemes, particularly when these are redundant. While this is also true of children acquiring their first language, and human communication generally, the tendency to focus on more content-full morphemes may be particularly pronounced in adult L2 learners who are often pushed to process complex utterances before their grammatical systems have become well entrenched. In line with this, a number of studies have shown that function words and inflectional morphology are particularly difficult for second language learners (White 2003; McDonald 2006; Hopp 2010, 2013). This lead Dąbrowska (2019) to hypothesize that traditional GJTs, which focus on the more “decorative” aspects of grammar, may underestimate L2 learners’ morphosyntactic abilities, and that tasks assessing grammatical comprehension may reveal areas of relative strength. The main purpose of this study is to test this hypothesis by directly comparing the same group of L2 learners on tasks tapping both “functional” and “decorative” grammar. To facilitate comparisons with earlier research, we use the same stimuli as earlier studies: specifically, we use a shortened version of the GJT task developed by Johnson and Newport (1989) to test “decorative” grammar, and Dąbrowska’s (2018, 2019) “Words and Sentences” test to assess mastery of “functional” grammar. The testing procedure was also very similar (see below).

In addition, we examine the effect of mode of presentation (spoken vs. written) of the stimuli for the grammaticality judgment task, as well as the context of learning (foreign language classroom vs. immersion in an L2-speaking community). The mode of presentation is relevant because it is possible that adult L2 learners’ problems on spoken grammaticality judgment tasks (sGJTs) are partly due to difficulties with processing the speech signal rather than grammar *per se*. In order to differentiate between sentences such as (1–3) and their grammatical counterparts, the participant must be able to determine whether the relevant grammatical marker (the alveolar fricative for the third person singular present, the alveolar plosive for the past tense, and an unstressed schwa in the case of the indefinite article) is present or absent. In rapid speech, these markers are of very short duration and easy to miss by a phonological system which is not optimized for the processing of the target language. Phonological factors could also be responsible for non-native speakers’ difficulties in example (4): for example, it is possible that some participants who judged the sentence as grammatical thought that the verb was *loved (/lʌvd/)*, not *laughed (/lɑ:ft/)*.

There is some research suggesting that sGJTs may indeed underestimate late L2 learners’ grammatical abilities. A recent meta-analysis of studies investigating age effects in second language ultimate attainment (Qureshi, 2016) reports that differences between early and later learners were greater in studies which used auditory as opposed to visual stimuli (Cohen’s *d* of 0.41 vs. 0.55), particularly when the judgments were timed (*d* = 0.45 vs. 0.85). Furthermore, a number of studies (e.g., Johnson, 1992; Bialystok and Miller, 1999; Jia et al., 2002; Clahsen et al., 2010; Spada et al., 2015; Shiu et al., 2018) have found that L2 learners performed better on written than on sGJTs. However, a recent review (Plonsky et al., 2019) suggests that the effect is weak and inconsistent, with some studies reporting an advantage for spoken stimuli.

It is also important to note that even if late L2 learners do in fact perform less well on sGJTs, this does not necessarily mean that such tasks underestimate their grammatical abilities in comparison with younger L2 learners or native speakers: if the latter two groups showed modality effects similar to those found in late learners, the use of auditory stimuli would be relatively unproblematic. Of the six studies cited in the preceding paragraph which reported original findings, five report results for native speaker controls. One study (Murphy, 1997) found that both native and non-native speakers performed better in the written condition, with no interaction between language background and modality. The remaining five studies report no significant differences between performance on spoken and written stimuli for the native speaker group. Since the scores achieved by native speakers in most of these studies are very high, this could be due to ceiling effects. It is worth noting, however, that in three studies (Johnson, 1992; Jia et al., 2002; Spada et al., 2015), native speakers performed slightly better in the spoken condition. The native participants tested by Bialystok and Miller (1999) achieved exactly the same score in both conditions; and those tested by Clahsen et al. (2010) performed marginally better in the written condition, with a difference of just one percentage point.

Thus, while there is some data suggesting that sGJTs may underestimate late L2 learners’ grammatical abilities, the evidence is far from conclusive. Further research is needed to determine whether or not performance on grammaticality judgment tasks depends on mode of presentation, and whether mode of presentation interacts with language background. In order to investigate these questions, we presented the same stimuli both in speech and in writing.

In short, the study described here was designed to test two predictions:

1. The differences between native and non-native speakers will be larger on the GJT task than on the picture selection task (and, as a corollary, more late L2 learners will perform within the native speaker range on the picture selection task);
2. L2 speakers will perform better on the GJT when the stimuli are presented in writing; for native speakers, the difference will be much smaller or non-existent.

Earlier research found robust relationships between education and performance on tasks tapping grammatical comprehension in native speakers (Dąbrowska, 1997, 2008, 2018; Chipere, 2001; Dąbrowska and Street, 2006; Street and Dąbrowska, 2010, 2014; Street, 2017). There is also a considerable amount of evidence suggesting that adult L2 learners with higher educational attainment tend to be more successful than less educated learners (Hakuta et al., 2003; Tarone et al., 2007; Janko et al., 2019). In addition, age at testing may affect the performance of native and non-native speakers in different ways (Dąbrowska, 2019). Therefore, to avoid confounds due to group differences in age or education, it is necessary to control for these variables. To make this possible, we decided to administer our experiment online, which allowed testing a much larger number of participants than would otherwise have been possible, and, consequently, to select a sample which was well matched on these variables. Although there is still some skepticism about the reliability of results obtained using web-based methods, a number of studies have shown that such results are very similar to those obtained in the lab (see, for example, Gosling et al., 2004; Reips and Birnbaum, 2011; Germine et al., 2012; Reimers and Stewart, 2015), even when these involve reaction times of only a few hundred milliseconds (Hilbig, 2016).

## Materials and Methods

### Participants

We recruited 1,555 participants through various internet fora, personal contacts, and an online linguistics course taught by the first author. Of these 1,555 participants, we excluded:

1. 650 who did not complete all three experimental tasks;
2. 189 who were either younger than 17 or older than 65 years of age; and
3. 10 who did not engage with one or more tasks (more than 10 consecutive timeouts), or where a hardware misfunction was suspected (a timeout on every other trial; the timeout criteria are explained below).

This left 706 participants, including 340 native speakers of English, 45 adult immersion learners (non-native speakers of English who had lived for at least 36 months in an English-speaking country and whose age of arrival in such a country was 17 or above), 247 classroom learners (non-native speakers who learned English at school who spent no more than 6 months in an English-speaking country), and 74 non-native speakers who could not be assigned to any of the above groups.

The participants ranged widely in age, education, and language background. Since those variables are known to affect performance of both native and non-native speakers, for the purposes of this study we selected a smaller but more balanced sample. Each of the 45 immersion learners was matched with one native speaker and one classroom learner. Native speakers and immersion learners were matched for education, age and, when possible, gender. Classroom learners and immersion learners were matched for education, age and, when possible, gender and native language. When an exact language match was not possible, participants with a native language from the same family were matched (e.g., Serbian and Slovak, both Slavic; Spanish and Italian, both Romance). The majority of the L2 participants (40 of the classroom learners and 41 of the immersion learners) were native speakers of languages belonging to the Indo-European family (see Supplementary Material A).The details of the participants’ age, education, and, for the non-native speakers, age of first exposure and estimated amount of English language instruction are given in Table 1. The mean age of arrival for immersion learners was 27.1 (SD 7.1, range 17–45) and the mean length of residence 13 years (SD 10, range 3–44).

**Table 1:** Demographic information on the matched sample.

Language group		Age	Education	Age of first exposure	EFL classroom hours
Classroom learners	M	43	16.0	11	2,220
	SD	11.7	1.9	7.24	2,008
	min-max	21–63	12–20	4–55	111–9,288						
Immersion learners	M	45	15.9	12	2,149
	SD	11.3	1.9	6.92	2,585
	min-max	26–65	12–20	4–38	0–12,492						
Native speakers	M	45	16.0		
	SD	11.6	1.8	from birth	NA
	min-max	26–65	12–20		

### Materials

#### The Grammaticality Judgment Tasks

Both grammaticality judgment tasks were based on the stimuli used in DeKeyser (2000) study, which in turn used a slightly modified version of the stimuli developed by Johnson and Newport (1989). However, because the participants were to complete three tasks, we selected 80 of the 200 items used by DeKeyser (2000), making sure that each of the 28 subcategories used in that study was represented in similar proportions as in the original test (see Supplementary Material B for details). Half of the stimuli (40) were grammatical and half (40) were ungrammatical. Both tasks were divided into two blocks of 40 items each; grammatical and ungrammatical versions of the same item were presented in different blocks and, within each task, the item order was randomized separately for each participant. The same stimuli were used in both versions of the task. The stimuli for the sGJT were recorded by a male native speaker of British English.

#### The Picture Selection Task

**Figure 1:**
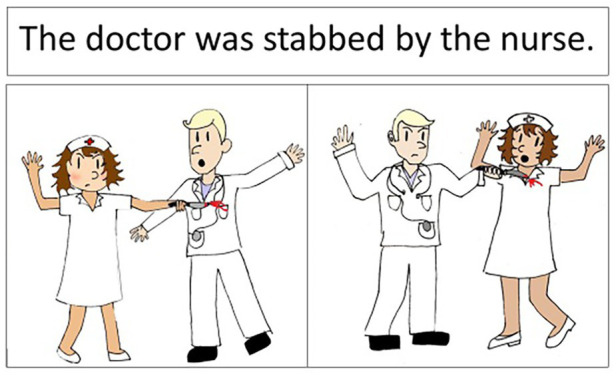
An example of an item from the picture selection task.

The picture selection task was an online version of Dąbrowska’s (2018, 2019) Pictures and Sentences Test. Each item in the test consists of a sentence and two pictures (see Figure 1 for an example) and the participant’s task is to choose the picture that goes with the sentence. There are 80 items representing 10 different construction types (see Supplementary Material C for details). The items are divided into eight blocks, each containing one token of each construction presented in random order.

### Procedure

All three tasks were administered online using PsyToolkit (Stoet, 2010, 2016), which recorded the participants’ responses as well as reaction times. The participants first completed a background questionnaire, which collected information about age, gender, education, occupation, and linguistic history. This was followed by the sGJT, PST, and the written grammaticality judgment task (wGJT). The sGJT was administered first since, to the extent that participants’ difficulties are (partly) attributable to the ability to perceive the presence of a particular morpheme in the spoken modality, seeing the same sentence in written form could affect performance with spoken stimuli, while an effect in the opposite direction is extremely unlikely with participants who are skilled readers.

The procedure for the sGJT closely followed that used by DeKeyser (2000) and Johnson and Newport (1989). Each item was played twice with a 2 s pause between repetitions. After the second presentation of the stimulus, participants were asked to press G if the sentence was grammatical, or U if it was ungrammatical. Participants were asked to respond as quickly and accurately as possible. If a participant did not respond within 8 s from the onset of the second presentation of the stimulus (i.e., 10 s from the offset of the first presentation), the trial timed out. There was a short break after the first 40 items.

During the picture selection task, the two pictures and a written sentence were presented at the same time. Participants responded by clicking on a radio button corresponding to the target picture. The next trial began as soon as the participant responded. Since the task involved the processing of complex visual stimuli as well as the target sentence, there was no timeout for this task.

The procedure for the wGJT was the same as for the spoken version of the task except that the stimuli were presented in writing. If a participant did not respond within 10 s from the onset of the trial, the trial timed out and the next trial began.

## Results

**Figure 2:**
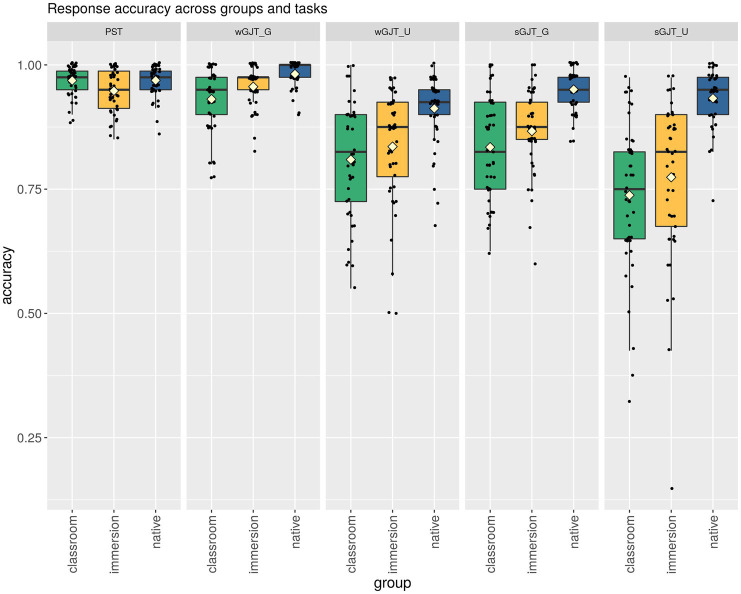
The distribution of scores across groups and tasks. The boxes contain 50% of the scores (IQR), the whiskers show Q3 + 1.5 * IQR and Q1–1.5 * IQR, respectively. The yellow diamonds indicate group means; the horizontal lines indicate the medians. The dots represent individual participants.

Performance on all three tasks is summarized in Table 2 and Figure 2. Since some studies (e.g., Murphy, 1997; Bialystok and Miller, 1999) found interactions between modality and grammaticality, we report performance for grammatical (G) and ungrammatical (U) items separately, and also model them separately in the following analyses. The matched sample summary data set (which includes background variables), the data set in long format (which was used for the model), and their descriptions are provided in Supplementary Materials D–G.

**Table 2:** Mean proportions of correct responses (and SDs) by group and task.

Language group	PST	wGJT	sGJT	wGJT_G	sGJT_G	wGJT_U	sGJT_U
Classroom learners	0.97 (0.03)	0.87 (0.08)	0.79 (0.11)	0.93 (0.06)	0.83 (0.10)	0.81 (0.12)	0.74 (0.15)
Immersion learners	0.95 (0.04)	0.90 (0.07)	0.82 (0.10)	0.96 (0.04)	0.87 (0.09)	0.84 (0.12)	0.77 (0.16)
Native speakers	0.97 (0.03)	0.95 (0.04)	0.94 (0.03)	0.98 (0.03)	0.95 (0.04)	0.91 (0.07)	0.93 (0.06)

In order to estimate the effect of language background on performance in the three tasks, we fitted a logistic regression model to predict the proportion of correct responses on the basis of speaker group, task, and (for the two GJTs) grammaticality. The model was fitted using Bayesian methods (Markov Chain Monte Carlo sampling) with Stan (Carpenter et al., 2017) with the *brms* package (Bürkner, 2017, 2018) in R (R Core Team, 2016). The model formula is given in (5):

5. response ~ task + group + task:group + task:group:taskident:gramm +

(1 | item) +

(1 | task:participant)

The model includes task and group as population-level (fixed) effects, with an interaction between task and group, and a nested interaction between task, group, and grammaticality for wGJT and sGJT only (the predictor “taskident” is a dummy variable set to “1” for the two GJTs and to “0” for the PST, ensuring that the three-way interaction only applies to the two GJTs). In addition to the population-level effects, the model includes the following group-level (random) effects: varying intercepts for individual items and for participants per task, controlling for effects of individual items and for effects of individual participants across the three tasks. The R code of the analysis is provided in Supplementary Material H.

**Figure 3:**
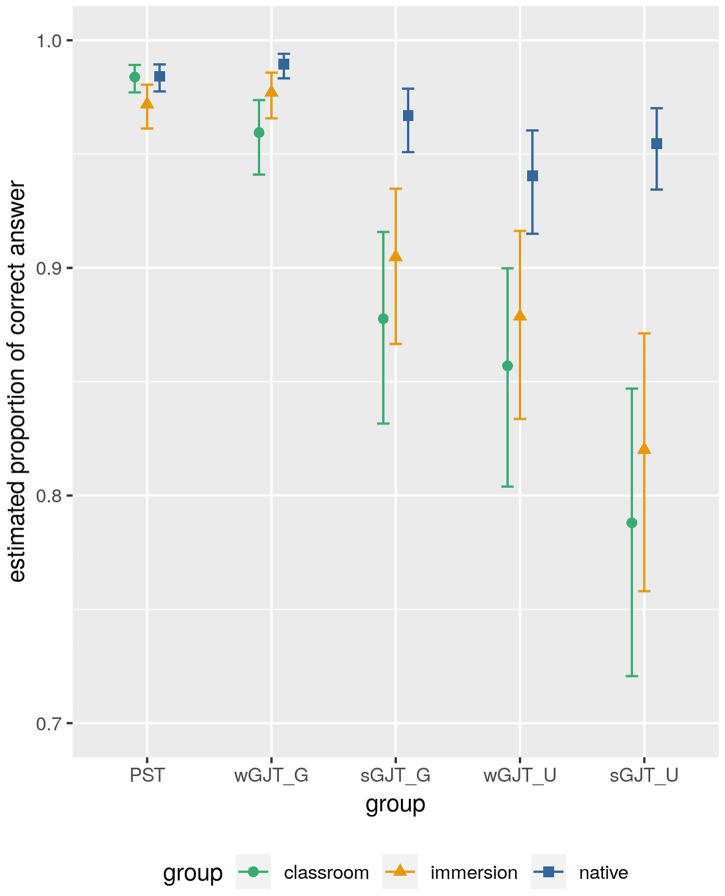
Estimated proportions of correct responses across speaker groups and tasks. The point estimates correspond to the mean of the probability density (i.e., the most likely estimates), and the whiskers indicate their 95% credible intervals.

A Bayesian model does not estimate a single best underlying coefficient (proportion of correct responses in our case) whose probability would then be evaluated against the null hypothesis. Instead, the estimated proportion of correct responses is modeled directly as a probability function: many different coefficients are possible, but they are not equally likely on the basis of prior assumptions (weakly informative prior were used here) and the data observed. Thus, modeling coefficients as probability functions makes it possible to interpret our degree of certainty about the real proportions of correct answers in a direct way and not against the null hypothesis. Table 3 and Figure 3 show the estimated proportions of correct responses per group and task as well as their credible intervals (CIs). The CI corresponds to the 95% density interval for the response estimate. In other words, given the data and the model, we can be 95% certain that the underlying proportions of correct responses in each of the 15 group and task combinations lie within the intervals shown. Further details about model coefficients and performance are provided in Supplementary Material I.

**Table 3:** Estimated probabilities of correct responses with 95% credible intervals (CIs) per task and group.

	Natives		Immersion		Classroom	
	Estimate	Q2.5–Q97.5	Estimate	Q2.5–Q97.5	Estimate	Q2.5–Q97.5
PST	0.98	0.98–0.99	0.97	0.96–0.98	0.98	0.98–0.99
wGJT_G	0.99	0.98–0.99	0.98	0.97–0.99	0.96	0.94–0.97
sGJT_G	0.97	0.95–0.98	0.90	0.87–0.94	0.88	0.83–0.92
wGJT_U	0.94	0.92–0.96	0.88	0.83–0.92	0.86	0.80–0.90
sGJT_U	0.96	0.93–0.97	0.82	0.76–0.87	0.79	0.72–0.85

The estimated scores shown in Table 3 and Figure 3 confirm the impression given by the descriptive statistics in Table 2, with the following two main trends: performance increases from the sGJT to the wGJT to the PST (with grammatical items of the wGJT and the PST showing virtually identical scores), and for the two GJTs, performance on grammatical items is better than on the ungrammatical items across all three groups. Comparing native and non-native speakers across the three tasks reveals clear interactions between task and group. In the PST, the CIs of all groups overlap, meaning that there are no real group differences. In the wGJT, we see that classroom learners have clearly lower scores than native speakers (the CIs do not overlap), while immersion learners performed at an intermediate level. In the sGJT, the estimates show pronounced differences between the classroom and immersion learners on the one hand and native speakers on the other. Finally, there is an interaction between modality and grammaticality: in the wGJT all three groups show better performance on grammatical sentences than on ungrammatical sentences, while in the sGJT, the differences are smaller and the CIs overlap, indicating that we cannot be certain that the difference is real.

It is important to note that the lack of real group differences for the PST task is not attributable to ceiling effects: as shown in Figure 2, there is considerable variation in scores in all three groups in all tasks, including the PST task – and as a result, considerable overlap between groups. To investigate this overlap further, we counted the number of participants in each group whose performance fell within the normal native speaker range. For this analysis, we adopted a widely used definition of “normal” performance, i.e., within two SDs of the mean. The mean and the SD were computed on the full native speaker sample (i.e., 340 speakers). The results of this analysis are presented in Table 4.

**Table 4:** Number (and percentage) of participants whose accuracy score fell within the normal native speaker range (±2 SDs) by group and task.

Language group	PST (71–80)	wGJT (70–80)	sGJT (69–80)
Classroom learners	41 (91.1%)	29 (64.4%)	15 (33.3%)
Immersion learners	37 (82.2%)	37 (82.2%)	21 (46.7%)
Native speakers	43 (95.6%)	42 (93.3%)	44 (99.8%)

The figures in the headers indicate the minimum and maximum values of the normal native speaker range for each task, based on the full sample of 340 native speakers.

In a normally distributed data set, approximately 95% of the scores should fall within two SDs of the mean, and, as we can see from the table, the figures for the native speakers in the matched sample are very close to this predicted value. More interestingly, for both the wGJT and the PST, the majority of the participants in non-native groups scored within the normal native speaker range. For the sGJT, the amount of overlap was smaller, but even here, 33% of the classroom learners and 47% of the immersion learners scored within the normal native range.

## Discussion

This section is organized as follows: firstly, we discuss our results in the context of the two predictions made in the introductory section. Secondly, since our results appear to contradict those obtained in some prior research, we present a detailed comparison with four earlier studies which used the same GJT test as we did. We conclude by discussing two important theoretical issues: whether adult L2 learning really is “defective”, and why “decorative” grammar is particularly difficult for adult learners.

### Prediction 1: Larger Difference Between Native and Non-Native Groups on the GJTs Than on PST

Our first prediction was that the differences between native and non-native speakers would be larger on grammaticality judgment tasks compared to the picture selection task. This was confirmed. There were relatively large differences between groups in the predicted direction for both grammaticality judgment tasks. For the picture selection task, however, the difference was much smaller with a substantial amount of overlap between the credible intervals for the model estimates, suggesting no real differences between groups. We discuss the implications of these findings in section “Is adult second language acquisition ‘defective’?”.

### Prediction 2: Larger Difference Between Native and Non-Native Groups on sGJT Than wGJT

Our second research question was whether scores on the GJT differ depending on mode of presentation (spoken vs. written). As predicted, both L2 groups achieved considerably higher scores on the wGJT than on the sGJT; this was true for both grammatical and ungrammatical sentences. The native speakers also showed a slight advantage for the written modality with grammatical sentences (in other words they were slightly more likely to accept a grammatical sentence when it was written than when it was spoken). With ungrammatical sentences, in contrast, they showed no real difference between modalities. The overall scores for native speakers in the spoken and written condition were virtually identical (94.1 and 94.9%, i.e., a difference of less than 1%). This contrasts with the relatively large differences observed in the non-native groups (8%).

As a result, the difference in performance between native and non-native speakers was smaller on the written task, and there was more overlap in scores between the groups. This effect was observed in spite of the fact that in the sGJT, participants were presented with the stimulus sentence two times and given a slightly longer response deadline (10 s from the *offset* of the first presentation in the case of the sGJT and 10 s in total for the wGJT). This confirms our suspicion that the large differences between native speakers and adult L2 learners observed in earlier studies are partially due to difficulties in processing spoken stimuli (which may be due to suboptimal phonological representations and/or less efficient processing) rather than morphosyntactic abilities *per se*. Thus, sGJTs may underestimate L2 speakers’ grammatical knowledge.

### Comparison With Earlier Research on Ultimate Attainment

Our results revealed the existence of significant differences between native speakers and both L2 learner groups in performance on both grammaticality judgment tasks but not on the picture selection task. However, even on the GJTs, a considerable number of L2 learners scored within the normal native speaker range. These findings appear to fly in the face of a large body of research on ultimate attainment in second language acquisition, which suggests that adult learners seldom, if ever, achieve native-like levels of proficiency (e.g., Coppieters, 1987; Johnson and Newport, 1989; Johnson et al., 1996; DeKeyser, 2000; Clahsen and Felser, 2006; Abrahamsson and Hyltenstam, 2009; DeKeyser et al., 2010; Granena and Long, 2013; Han, 2013). It is important, therefore, to examine the reasons for these differences. As explained earlier, our stimuli were based on those used by DeKeyser (2000), who in turn used a shortened version of the test developed by Johnson and Newport (1989). Two other studies (Birdsong and Molis, 2001; DeKeyser et al., 2010) used more or less the same stimuli. The participants tested by these researchers were all immersion learners, but they came from different L1 backgrounds: Korean and Japanese in the case of Johnson and Newport, Hungarian in the case of DeKeyser (2000), Russian in the case of DeKeyser et al. (2010), and Spanish in the case of Birdsong and Molis. In this section, we compare our results to those reported by these researchers.

All four studies focused primarily on the relationship between age of arrival and ultimate attainment, and therefore included a high proportion of participants with ages of arrival much lower than those examined in this paper. Furthermore, the sample tested by DeKeyser et al. (2010) also included participants with very high ages of arrival (up to 71). To allow for meaningful comparisons with our results, therefore, we include only data from the participants in the earlier studies who were aged between 17 and 45 at the time they arrived in an English-speaking country. This includes 23 speakers from the Johnson and Newport sample, 32 participants tested by Birdsong and Molis (2001), 42 participants tested by DeKeyser (2000), and 34 participants from the DeKeyser et al. (2010) sample. The data for Johnson and Newport, Birdsong and Molis, and DeKeyser (2000) were taken directly from these studies; those for DeKeyser et al. (2010) were obtained from the online supplementary materials published with Vanhove (2013) re-analysis of the original data set.

**Figure 4:**
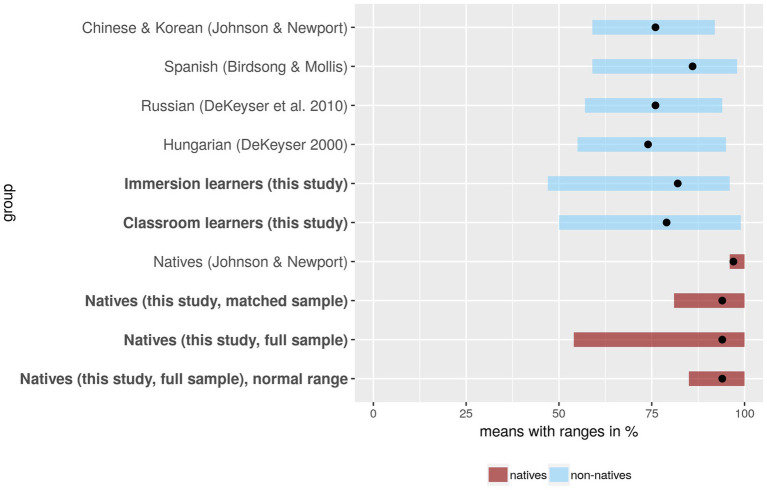
Performance on spoken grammaticality judgment task (sGJT) studies in five studies using the stimuli from Johnson and Newport (1989). The last line provides the normal range (±2 SDs from the mean) for the full sample. All other ranges are defined by minimum and maximum scores.

The means and ranges for the four studies as well as for our data are summarized in Figure 4. It is clear from the figure that the results for immersion learners are remarkably consistent across the five data sets. The mean proportions of target responses in the two samples of speakers of non-Indo-European L1s (Johnson and Newport, 1989; DeKeyser, 2000) and DeKeyser et al. (2010), who tested Russian speakers, were somewhat lower (74, 76, and 76%, respectively), while the Spanish speakers tested by Birdsong and Molis (2001) obtained somewhat higher scores (mean 86%). As explained earlier, the majority of our participants were speakers of Indo-European languages (mostly Romance and Germanic), and their mean score was 82%, slightly above the mean for the Russian speakers tested by DeKeyser et al. (2010) and slightly below Birdsong and Molis’ Spanish speakers. The ranges for the non-native speakers are also remarkably similar, with studies involving larger samples reporting, predictably, somewhat wider ranges.

Our results for L2 learners, therefore, are perfectly in line with those reported in the earlier studies; the difference is in results for native speaker controls. Only one of these four studies, namely Johnson and Newport (1989), actually collected data from native speakers; the other three focused simply on the relationship between age of arrival and performance on the grammaticality judgment task – an issue we will return to later. Johnson and Newport (1989) report that their native controls supplied the target answer on 97% of the trials on average, with scores ranging from 96 to 100%; they were thus clearly at ceiling. In our study, the mean accuracy rate for the native speakers in the matched sample (*N* = 45) was somewhat lower (94%), and the range was considerably wider (from 81 to 100%). For the full sample (*N* = 340), the mean was likewise 94%, and the range from 54 to 100%. The overall range is not very informative, since it is defined by extreme scores, which could be unrepresentative. Indeed, the participant with the lowest score in the native group is clearly an outlier: the next lowest score was 80%. For this reason, in order to assess the amount of overlap between native and non-native speakers, we used the normal native speaker range, defined as ±2 SDs from the mean computed for the full sample (shown as the last line in Figure 4). For the sGJT, the normal native speaker range was from 86 to 100% (raw scores from 69 to 80).

Why should there be such a large difference between the two studies? Johnson and Newport’s control group consisted of 23 native speakers of English^1^. The authors provide no further details about this sample. However, they do indicate that the non-native participants were university students, professors, and research associates, and presumably the native controls were recruited from the same population: in other words, they were likely to have been highly educated. Our native group was larger and more heterogeneous in terms of schooling, with the number of years spent in full time education ranging from 12 (i.e., secondary school diploma) to 20 (i.e., a completed PhD), and a mean of 16 years. Our sample, therefore, provides a more realistic estimate of the amount of variation found in native speakers.

As previously shown by Andringa (2014) and Dąbrowska (2019), the proportion of late L2 learners performing within the native speaker range depends on the composition of the native speaker sample: if we use a demographically more varied native speaker sample, there is more overlap. It is worth noting in this connection that our native sample, though more representative than that used by Johnson and Newport, probably also underestimates the amount of variation in native speakers. This is evident when we compare the results for the picture selection task for our native speakers with those reported by Dąbrowska (2018). As explained in the introductory section, Dąbrowska (2018) used a sample whose composition roughly reflected the demographic structure of the UK population. The mean number of years spent in full-time education for Dąbrowska’s sample was 13.7 (range: 10–21), while for the immersion sample used in this study, the corresponding figure was 15.9 (range 12–20). As shown in Table 5, our native participants showed considerably less variation in scores than those tested by Dąbrowska (2018), and their group mean was about one SD higher.

**Table 5:** Comparison of two native speaker groups on the picture selection task (PST).

	Mean	SD	Range	Normal range
Dąbrowska (2018)	93	5	73–100	83–100
This study	97	4	83–100	89–100

The discussion so far has focused on whether, and how often, adult L2 learners can achieve levels of performance comparable to native speakers on tasks measuring grammatical knowledge. Most critical period studies ask a different question, namely, whether there is a negative correlation between age of arrival and performance on some linguistic task, and whether there is evidence of a discontinuity in the relationship. We believe that this question is less relevant to research seeking to establish whether or not there is a critical period for learning a second language, for two reasons.

First, in immersion settings, there is a nearly perfect correlation between AoA and the amount of native input (Flege, 2019): early arrivals nearly always have more years of schooling in the L2 and more friends who are native speakers, are more likely to be married to native speakers, and so on. This means that the correlation between AoA and grammatical proficiency could be spurious, with the real cause being the amount of input, or the amount of native speaker input, available to the learner.

Secondly, immersion in the L2 at an early age very often results in incomplete acquisition and/or loss of proficiency in the L1. This is demonstrated by a growing body of research on first language development in heritage language speakers (see e.g., Polinsky and Kagan, 2007; Montrul, 2008; Romanova, 2008; Kim et al., 2010; Benmamoun et al., 2013). Heritage language speakers are speakers who learn a minority language at home as children and the majority language at school and (usually) in the playground. In early childhood, they are typically either monolingual in the heritage language or bilingual but dominant in the heritage language. By middle childhood, most are balanced bilinguals; by the time they become teenagers, they are typically dominant in the majority language; and by adulthood, some speakers with a heritage language background are effectively monolingual. Heritage language speakers typically have relatively good pronunciation, but their grammars are often incompletely developed (compared to monolingual speakers) and sometimes deviant: in fact, their speech is similar in many ways to the output of adult L2 learners (Montrul, 2008; Kim et al., 2010). Adult L2 learners sometimes also experience some degree of L1 attrition, but to a much smaller extent, and only after a long period of non-exposure. This means that studies comparing child and adult L2 learners effectively demonstrate that bilinguals are more native-like in their dominant language than in their weaker language, which is hardly surprising.

### Is Adult Second Language Acquisition “Defective”?

As we have seen, for all three tasks, a large proportion (from 33 to 91%) of our L2 learners performed within the native speaker range. This is remarkable, considering the amount of exposure to English of our non-native participants. Hartshorne et al. (2018) report the results of a study, which used a similar methodology to ours (online experiment involving grammaticality judgment and picture selection items). Their experiment used a shorter test, but a much larger sample, including 246,00,000 monolingual native speakers. Perhaps the most surprising finding of the study was that in the native speaker group, performance increased up to about age 30. Assuming that a typical monolingual native speaker is exposed to their language for 8 h per day (a rather conservative estimate), by age 30 she/he will have had 87,600 h of exposure (8 h × 365 days × 30 years). Our classroom learners, in contrast, had, on average, only 2,220 h of instruction (although of course they are likely to have also experienced some English outside the classroom), and the immersion learners had, on average, 2,214 h of instruction and 10 years of residence in an English speaking country (during which many were likely to have continued to use the L1 a considerable proportion of the time). It is also worth noting that adult L2 learners, whether in instructional or naturalistic settings, are typically exposed to a high proportion of non-native, and hence often deviant, input. Given these differences in input, and the amount of overlap between the native and non-native groups, adult L2 learning should be regarded as extremely efficient rather than defective.

An important clarification is in order here. We are not claiming that our participants were native-like in every respect. As explained earlier, the majority took part in a MOOC taught by the first author. Although we cannot match individual participants’ performance with their contributions to online discussions, it is clear that the written output of a large number non-native contributors was far from native-like; as a rough estimate, most of our L2 participants’ knowledge of English was probably at CEFR level B2 to C1.

This leads us to an important point. It does not make sense, in our view, to speak of a critical period for “language”, or even “grammar” as such. Adult L2 learners are very successful in acquiring some aspects of the target language: for example, “functional” grammar is clearly an area of strength. It is equally clear that some aspects of grammar (in particular, tense and agreement morphology and idiosyncratic properties of specific individual lexical items) are difficult for many adult learners. Studies investigating age effects in acquisition often focus on those aspects of the L2 grammar, which are known to be difficult for learners with a particular L1 or for adult L2 learners generally. Although this is a reasonable strategy if one is interested in discovering differences in the outcome of L1 and L2 acquisition, ignoring aspects of grammar which are acquired relatively quickly by adult learners gives us a very biased view of L2 attainment.

Another important point to bear in mind is that the fact that a high proportion of L2 learners achieved scores within the native speaker range does not necessarily entail that they processed the sentences in the same way as native speakers. All three of our experimental tasks were off-line and participants were given a relatively long time to respond. This means that our L2 participants could, in principle, have accessed explicit knowledge about language, whereas this is unlikely to have been the case for native speakers, since L1 grammatical knowledge is known to be mostly tacit. One way to address this issue would be by examining whether the amount of time available to process the stimulus affects native and non-native speakers differently (an issue that our group is currently investigating).

### What Do L2 Learners’ Strengths and Weaknesses Tell Us About Language Learning?

As we have seen, the differences between native speakers and both non-native groups were much larger for “decorative” than for “functional” grammar. Why should this be the case, and conversely, why did adult learners do so well on the “functional” grammar task? Before we turn to this issue, some clarifications are in order. First, as already indicated earlier, we are *not* suggesting that there are two different types of grammar, one of which carries meaning while the other does not. The “functional”-“decorative” distinction is a continuum: some aspects of grammar clearly contribute to the meaning conveyed by an utterance, while for others, the contribution may be relatively modest and/or redundant; and many fall at some point in between. Secondly, the term “decorative” is not meant to be deprecatory. “Decorative” grammar, by definition, has a comparatively low communicative load, and therefore errors involving it typically do not lead to communication failure – but occasionally they do. Furthermore, speakers who omit grammatical morphemes or use them inappropriately are likely to be perceived as less intelligent or less competent by native speakers (Llurda, 1993; Johnson and Jenks, 1994). Finally, “decorative” grammar is interesting from a theoretical point of view precisely because it poses particular difficulties for L2 learners.

Why, then, did L2 learners do so well on the “functional” grammar test and relatively less well on the grammaticality judgment tasks? Although the results reported here do not speak to this issue, we can glean some hints from earlier research. A number of L1 acquisition studies have found robust correlations between measures of nonverbal intelligence and performance on grammatical comprehension tests such as the TROG (Roth et al., 2002; van der Schuit et al., 2011; Gallinat and Spaulding, 2014; West et al., 2017). Furthermore, there is some evidence of a relationship between language aptitude (as measured by *foreign* language aptitude tests) and grammatical proficiency in the L1. Thus, Dąbrowska (2018) found a moderately strong correlation (0.45) between the Language Analysis subtest of the Pimsleur Language Aptitude Battery and native speakers’ performance on the “Pictures and Sentences” test. Even more striking are the results reported by Skehan and Ducroquet (1988), who found significant correlations (some as high as 0.52) between scores on language aptitude tests obtained from a group of 14-year-olds and various measures of L1 development taken from the same participants in early childhood (between the ages of 3;3 and 5;5). This suggests that “functional” grammar may depend to some extent on explicit reasoning and IQ – abilities, which are much better developed in adults than in children.

Turning now to the second part of the questions – why “decorative” grammar is relatively difficult for adult learners – we should first note that grammatical morphemes with low functional load and low perceptual salience have been found to be generally vulnerable, not just in L2 learning, but also in aphasia, developmental language disorder, and even in normal adults under pressure (Blackwell and Bates, 1995; McDonald, 2006). They are also difficult for children. Young children acquiring English as a first language often omit function words and grammatical inflections in obligatory contexts, as shown in the following examples taken from the Sarah corpus (Brown, 1973), recorded when Sarah was 2;9.20.

1.
  - *two broom // two horsie*.
  - *I ride horsie*.
  - *he cry // he crying*.

Importantly, just like L2 learners, children acquiring English as their first language continue to produce such “telegraphic” utterances even after they have become productive with the relevant morphemes. In other words, they go through a period of inconsistent use, when a given grammatical morpheme is supplied on some occasions but not on others. For example, the same transcript from the Sarah corpus also contains the utterances in (6), where the relevant grammatical morphemes (plurals, determiners, tenses, and agreement markers) are supplied correctly:

1.
  - *I wash my hands // I want two pennies // I wanna play piggies*.
  - *a nickel // a big circus // lift a latch // push the button*.
  - *he cried // she cried // he cries // he laughs*.

This period of inconsistent use can be quite long. English-speaking children typically start using the regular past tense ending about the age of 2;4 (2 years and 4 months), and start using it productively (as evidenced by overgeneralization errors) a few months later. The proportion of correctly marked forms increases steadily, reaching adult-like levels (i.e., virtually 100%) by about 5;6 according to some studies (Rice et al., 1998) and as late as age 10 according to others (Marchman et al., 1999; van der Lely and Ullman, 2001). Assuming that children are exposed to language for 8 h per day on average (which is a rather conservative estimate), this means that they need between 8,760 (3 × 365 × 8) and 23,360 (8 × 365 × 8) hours of exposure before they reliably supply the regular inflection.

However, this is only part of the story, since L1 learners eventually stop producing such errors, while in many L2 learners they persist even at very advanced levels, as illustrated by the following examples, taken from written work produced by a postgraduate Chinese student studying at a British university:

1.
  - These levels, therefore, are necessary mechanism for such constructions. [article omission]
  - Gries and his colleagues …. argue that the collostructional strength in their collostructional analysis reveals various degree of verb-construction semantic compatibility. [plural morpheme omission]

Some researchers (e.g., Paradis, 2004; DeKeyser et al., 2010; Granena and Long, 2013; Ullman, 2015) attribute the problems that adult L2 learners have to deficits in implicit learning. However, there is actually very little evidence supporting the claim that adults are less efficient at implicit learning than children. Most studies which demonstrate age-related decline involved comparisons of young and middle-aged or elderly adults (Howard and Howard, 2013), and are thus irrelevant to the age effects debate in SLA research. There are very few studies which compared children and young adults. Two of these (Thomas et al., 2004; Lukács and Kemény, 2015) found that adults learned better. To the best of our knowledge, only one study (Janacsek et al., 2012) found that younger participants (aged 12 or below) learned more than teenagers and adults. However, the difference showed up only when comparing reaction times on low‐ and high-probability sequences (and not on accuracy and not on Z-transformed RT measures). More importantly, a statistically significant difference between groups does not entail a fundamental difference: to demonstrate a fundamental difference, it would be necessary to show that there is very little or no overlap between learning scores in children compared to adults.

A more promising explanation for L2 learners’ particular difficulties with “decorative” morphology is in terms of learned attention, an associative learning phenomenon whereby earlier learned cues attentionally block those that are experienced later (Ellis, 2008; Ellis and Sagarra, 2010, 2011; Ellis et al., 2014): for example, early experience with adverbial cues may block the acquisition of verbal tense morphology, and conversely, early experience with tense blocks the learning of adverbs. This also operates at a cross-linguistic level: thus, native speakers of languages with no tense morphology (such as Chinese) find it more difficult to learn tense than native speakers of languages that do mark tense.

Yet another explanation would be in terms of motivation. There is considerable evidence that individual differences in L2 attainment are partly due to motivation, and in particular, integrative motivation (see e.g., Masgoret and Gardner, 2003). Children may be better at learning “decorative” grammar because they are more focused on fitting in, while adult learners are more goal-directed, i.e., more focused on getting the message across, and hence attend more to those aspects of language which contribute to meaning. It is interesting to note that other aspects of language where adult learners often fail to reach native-like levels, viz. collocational choices and pronunciation, are, like “decorative” grammar, more relevant for displaying affiliation than for getting the message across (in the sense that a slight accent or a somewhat unidiomatic word choice will usually not impede communication, but immediately marks the speaker as an outsider).

## Conclusion

The grammars of adult L2 learners are often regarded as “incomplete” or “defective” in some way compared to those of native speakers. However, the results reported here show that a considerable proportion of adult learners can, and do, perform within the normal native speaker range on tests tapping grammatical knowledge, particularly in those aspects of grammar, which make a clear difference to meaning. Given the differences in the quantity and quality of the input that is typically available to L1 and L2 learners, as well as the other differences discussed in the preceding section (learned attention, motivation), it is simply wrong to think of L2 learning as “defective.” Even if there is a critical period, or critical periods, for certain aspects of language, we should not neglect the fact that adult L2 learners are extremely efficient learners in many other areas.

## Data Availability Statement

All datasets presented in this study are included in the article/Supplementary Material.

## Ethics Statement

The studies involving human participants were reviewed and approved by Humanities and Social Sciences Ethical Review Committee, University of Birmingham. Written informed consent for participation was not required for this study in accordance with the national legislation and the institutional requirements. The participants provided online informed consent to participate in this study.

## Author Contributions

ED designed the study, prepared the experimental tasks and collected the data. LM and LB processed the data. LB was responsible for statistical modeling. All authors contributed to drafting and revising the paper, and have approved the final version of the manuscript. All authors contributed to the article and approved the submitted version.

### Conflict of Interest

The authors declare that the research was conducted in the absence of any commercial or financial relationships that could be construed as a potential conflict of interest.

## Footnotes

^1^It is worth noting that Johnson and Newport actually tested 25 natives, but two were excluded, “one because the posttest interview revealed that he acquired English outside of the US, and one because she spoke a nonstandard dialect of English” (Johnson and Newport 1989, p. 70). It is not clear why this should matter, since the authors did not specify any inclusion criteria for native speakers, and one wonders whether the decision to exclude these two participants was made *post hoc*, possibly because they were outliers.
