# Supplementary material for: Is Adult Second Language Acquisition Defective?
Source: Front Psychol. 2020 Jul 30;11:1839. doi: 10.3389/fpsyg.2020.01839 (PMC7409517; doi:10.3389/fpsyg.2020.01839)
Supplement: Supplementary file 1 [file Data_Sheet_1.ZIP › Appendix G.DOCX]

**Appendix G: Description of the matched data set**

**Summary dataset (Appendix D “df-summary.csv”)**

matchid: identifies participants who were matched with each other

edu: number of years in full-time education

native: whether the participant is a native speaker of English

native_lang: the participants native language(s)

lang_subfamily: the subdivision within a language family to which the participant’s first language belongs

lang_family: the language family of the participant’s native language

first_exp: age at first exposure to English

eng_edu: estimated number of hours of instruction in English

LoR: length of residence in an English-speaking country in months

AoA: age of arrival in an English-speaking country

variety: variety of English spoken

group: experimental group (native, classroom learner or immersion learner)

score_sp: score on the spoken GJT

mean_rt_sp: mean reaction time on the spoken GJT

mean_wr: score on the written GJT

mean_rt_wr: mean reaction time on the written GJT

score_ps: score on the picture selection task

mean_rt_ps: mean reaction time on the picture selection task

**Long format (Appendix E “df-long.csv”, Appendix F “df-long.RDS”)**

participant: individual participant ID

response: "1" for correct response, "0" for incorrect response or time-out

task: task ("sGJT" for spoken GJT, "wGJT" for written

GJT, "PST" for picture selection task)

item: question item (1-80 for the two GJTs, P1-P80 for PST)

group: experimental group (native, classroom learner, immersion learner)

taskident: dummy variable for model with "1" for sGJT and wGJT, and "0" for PST

gramm: grammaticality of the question item ("G" for grammatical, "U" for

ungrammatical, "ps" for all PST items)
