# Supplementary material for: Is Adult Second Language Acquisition Defective?
Source: Front Psychol. 2020 Jul 30;11:1839. doi: 10.3389/fpsyg.2020.01839 (PMC7409517; doi:10.3389/fpsyg.2020.01839)
Supplement: Supplementary file 1 [file Data_Sheet_1.ZIP › List of supplemantary materials.docx]

**List of supplementary materials**

**Appendix A**: L1 background of the non-native participants

**Appendix B**: Stimuli for the grammaticality judgment tasks

**Appendix C**: Constructions tested in the Picture Selection Task

**Appendix D**: Matched sample summary data set (df-summary.csv)

**Appendix** **E**: Full data set in long format (df-long.csv)

**Appendix F:** Full data set in long format (df-long.RDS)

**Appendix G**: Descriptions of the datasets

**Appendix H**: R code used in the analysis (code.R)

**Appendix I**: Model coefficients and performance
