# Supplementary material for: Is Adult Second Language Acquisition Defective?
Source: Front Psychol. 2020 Jul 30;11:1839. doi: 10.3389/fpsyg.2020.01839 (PMC7409517; doi:10.3389/fpsyg.2020.01839)
Supplement: Supplementary file 1 [file Data_Sheet_1.ZIP › Appendix B.docx]

**Appendix B: Stimuli for the grammaticality judgment tasks**

| **Item** | **Grammatical variant** | **Ungrammatical variant** | **Category** | **Subcategory** |
| --- | --- | --- | --- | --- |
| 01 | Last night the old lady died in her sleep. | Last night the old lady die in her sleep. | Past Tense | Past tense marking omitted in obligatory context |
| 02 | Janie slept with her teddy bear last night. | Janie sleeped with her teddy bear last night. |  | Irregular verbs regularized |
| 03 | A bat flew into our attic last night. | A bat flewed into our attic last night. |  | Regular ending on irregular stem |
| 04 | Three boys played on the swings in the park. | Three boy played on the swings in the park. | Plural | Plural marking omitted in obligatory context |
| 05 | A shoe salesman sees many feet throughout the day. | A shoe salesman sees many foots throughout the day. |  | Irregular plurals regularized |
| 06 | I need to get some information about the train schedule. | I need to get some informations about the train schedule. |  | Mass nouns used with plural marker |
| 07 | John’s dog always waits for him at the corner. | John’s dog always wait for him at the corner. | Third-Person Singular | Third-person -s omitted in obligatory context |
| 08 | John can play the piano very well. | John can plays the piano very well. |  | Third-person -s marked on main verb after modals |
| 09 | Mary will go to Europe next year. | Mary will goes to Europe next year. |  | Third-person -s marked on main verb after modals |
| 10 | The little boy is speaking to a policeman. | The little boy is speak to a policeman. | Present Progressive | Progressive -ing omitted in obligatory context |
| 11 | Tom is working in his office right now. | Tom working in his office right now. |  | Progressive auxiliary omitted |
| 12 | Tom is reading a book in the bathtub. | Tom is reading book in the bathtub. | Determiners | Determiner omitted in obligatory context |
| 13 | Beauty is something that lasts forever. | The beauty is something that lasts forever. |  | Determiner used with abstract nouns |
| 14 | The men played basketball in the backyard. | The men played the basketball in the backyard. |  | Determiner used with abstract nouns |
| 15 | Peter made out the check but didn’t sign it. | Peter made out the check but didn’t sign. | Pronominaliza-tion | Pronoun omitted in obligatory context |
| 16 | Mary looked at the flowers but didn’t buy them. | Mary looked at the flowers but didn’t buy. |  | Pronoun omitted in obligatory context |
| 17 | The girl cut herself on a piece of glass. | The girl cut himself on a piece of glass. |  | Gender errors |
| 18 | Peter did not have any money on him. | Peter did not have any money on her. |  | Gender errors |
| 19 | The man climbed up the ladder carefully. | The man climbed the ladder up carefully. | Particle Movement | Phrasal verb separation not allowed |
| 20 | The new neighbours carried on a long conversation. | The new neighbours carried a long conversation on. |  | Phrasal verb separation not allowed |
| 21 | Kevin called Nancy up for a date. | Kevin called Nancy for a date up. |  | Phrasal verb separation allowed, but particle moved too far |
| 22 | Mary took her coat off quickly. | Mary took her coat quickly off. |  | Phrasal verb separation allowed, but particle moved too far |
| 23 | George says his prayers much too softly. | George says much too softly his prayers. | Subcategoriza-tion | Subcategorization |
| 24 | The little boys laughed at the clown. | The little boys laughed the clown. |  | Subcategorization |
| 25 | John told me that his wife was ill. | John said me that his wife was ill. |  | Subcategorization |
| 26 | The man allows his son to watch TV. | The man allows his son watch TV. |  | Subcategorization |
| 27 | The man lets his son watch TV. | The man lets his son to watch TV. |  | Subcategorization |
| 28 | The girls enjoy watching TV. | The girls enjoy to watch TV. |  | Subcategorization |
| 29 | Will Harry be blamed for the accident? | Will be Harry blamed for the accident? | Yes-No Questions | *aux Aux s[ . . . |
| 30 | Can the little girl ride a bicycle? | Can ride the little girl a bicycle? |  | *aux Verb s[ . . . |
| 31 | Is Sally waiting in the car? | Is waiting Sally in the car? |  | *aux Verb s[ . . . |
| 32 | Does John know the answer to that question? | Knows John the answer to that question? |  | *V s[… |
| 33 | Where did Arnie hunt last year? | Where did Arnie hunted last year? | Wh-Questions | Double tense marking |
| 34 | What is Martha bringing to the party? | What Martha is bringing to the party? |  | No aux inversion |
| 35 | Who do you meet at the park every day? | Who you meet at the park every day? |  | No aux |
| 36 | The man burned the dinner. | The dinner the man burned. | Word Order | S V DO order violated |
| 37 | The woman asked the policeman a question. | The woman the policeman asked a question. |  | S V IO DO order violated |
| 38 | The dog bites. | Bites the dog. |  | S V order violated |
| 39 | The students went to the movies. | The students to the movies went. |  | S V PP order violated |
| 40 | The student eats his meals quickly. | The student eats quickly his meals. |  | Adverb placement |
