# Supplementary material for: Is Adult Second Language Acquisition Defective?
Source: Front Psychol. 2020 Jul 30;11:1839. doi: 10.3389/fpsyg.2020.01839 (PMC7409517; doi:10.3389/fpsyg.2020.01839)
Supplement: Supplementary file 1 [file Data_Sheet_1.ZIP › Appendix C.docx]

**Appendix C: Constructions tested in the Picture Selection Task**

| **Construction** | **Example** |
| --- | --- |
| Active | The boy scratched the dancer. |
| Passive | The dancer was scratched by the boy. |
| Subject cleft | It was the boy that scratched the dancer. |
| Object cleft | It was the dancer that the boy scratched. |
| Subject relative | The boy was the one who scratched the dancer. |
| Object relative | The dancer was the one that the boy scratched. |
| Simple locative | The lamp is on the table. |
| Locative w/ quantifier | Every lamp is on a table. |
| Possessive locative w/ quantifier | Every table has a lamp on it. |
| Post-modifying prepositional phrase | The lamp on the table is white. |

The entire test is available from IRIS repository (<https://www.iris-database.org/iris/app/home/detail?id=york:935511>).
