# Supplementary material for: Is Adult Second Language Acquisition Defective?
Source: Front Psychol. 2020 Jul 30;11:1839. doi: 10.3389/fpsyg.2020.01839 (PMC7409517; doi:10.3389/fpsyg.2020.01839)
Supplement: Supplementary file 1 [file Data_Sheet_1.ZIP › Appendix A.docx]

**Appendix A: L1 background of the non-native participants**

| **Language Family / Genus** | **Native Language** | **Classroom Learners** | **Immersion Learners** |
| --- | --- | --- | --- |
| Arabic | Arabic | 1 | 1 |
| Finno-Ugric | Estonian | 2 | 1 |
|  | Hungarian | 1 | 1 |
| Greek | Greek | 2 | 3 |
| Indo-Aryan | Punjabi | 1 | 1 |
|  | Sinhalese | 0 | 1 |
| Germanic | Norwegian | 1 | 1 |
|  | Dutch | 2 | 0 |
|  | German | 5 | 7 |
| Romance | French | 1 | 8 |
|  | Italian | 4 | 3 |
|  | Portuguese | 8 | 2 |
|  | Romanian | 2 | 0 |
|  | Spanish | 7 | 8 |
| Slavic | Croatian | 1 | 0 |
|  | Polish | 3 | 4 |
|  | Serbian | 2 | 1 |
|  | Slovak | 1 | 2 |
| Thai | Thai | 1 | 1 |
