# Supplementary material for: Is Adult Second Language Acquisition Defective?
Source: Front Psychol. 2020 Jul 30;11:1839. doi: 10.3389/fpsyg.2020.01839 (PMC7409517; doi:10.3389/fpsyg.2020.01839)
Supplement: Supplementary file 1 [file Data_Sheet_1.ZIP › Appendix I.docx]

**Appendix I: Model coefficients and performance**

**Table I1**: Estimates for the population level effects in log-odds

| **Population-level effects** | **Estimate** | **Est.Error** | **L-95% CI** | **U-95% CI** |
| --- | --- | --- | --- | --- |
| Intercept | 2.96 | 1.69 | -0.3 | 6.34 |
| wGJT | 0.48 | 2.24 | -3.94 | 4.78 |
| PST | 1.18 | 1.69 | -2.2 | 4.41 |
| immersion | -0.61 | 2.01 | -4.5 | 3.3 |
| classroom | -0.72 | 2.05 | -4.83 | 3.23 |
| wGJT:immersion | 0.03 | 2.89 | -5.64 | 5.67 |
| PST:immersion | 0.02 | 2.01 | -3.88 | 3.94 |
| wGJT:classroom | -0.15 | 2.85 | -5.76 | 5.44 |
| PST:classroom | 0.7 | 2.05 | -3.23 | 4.82 |
| sGJT:native:G | 0.44 | 1.68 | -2.9 | 3.68 |
| wGJT:native:G | 1.15 | 2.09 | -2.89 | 5.28 |
| sGJT:immersion:G | -0.08 | 2.05 | -4.1 | 3.97 |
| wGJT:immersion:G | 0.92 | 2.49 | -3.92 | 5.78 |
| sGJT:classroom:G | -0.26 | 2.05 | -4.25 | 3.76 |
| wGJT:classroomG | 0.62 | 2.43 | -4.15 | 5.37 |
| sGJT:native:U | 0.11 | 1.68 | -3.25 | 3.35 |
| wGJT:native:U | -0.66 | 2.09 | -4.72 | 3.47 |
| sGJT:immersion:U | -0.82 | 2.05 | -4.83 | 3.23 |
| wGJT:immersion:U | -0.87 | 2.48 | -5.72 | 3.99 |
| sGJT:classroom:U | -0.92 | 2.05 | -4.9 | 3.08 |
| wGJT:classroom:U | -0.76 | 2.43 | -5.54 | 3.98 |

The model fully converged (Rhat=1 for all population and group-level effects). To evaluate the model, Bayes R^2^ was calculated following Gelman et al. (2019) and their supplementary materials (https://avehtari.github.io/bayes_R2/bayes_R2.html). The R^2^ value is 0.16 (CIs: 0.152, 0.174); such a relatively low R^2^ value is partially due to the vast majority of responses in the data are correct, meaning that a correct prediction without information is fairly easy, which the R^2^ penalized. A so-called confusion matrix between predicted and observed responses shows exactly this; the model slightly underestimates incorrect answers. Therefore, the overall accuracy of the model's predictions is very high (0.91), while a measure such as the kappa statistics (taking into account the "difficulty" of correct predictions without information only given the distribution of the responses) is very low (0.12) similarly to the R^2^ value.

**Table I2**: Confusion matrix between predicted and observed responses

|  | **observed** | |
| --- | --- | --- |
| **predicted** | **0** | **1** |
| **0** | 223 | 158 |
| **1** | 2710 | 29169 |
| **Acurracy** | 0.9111 | |
| **Kappa** | 0.1161 | |

Gelman, A., Goodrich, B., Gabry, J., and Vehtari, A. (2019) R-Squared for Bayesian Regression Models. *The American Statistician* 73, 307–9. doi: [10.1080/00031305.2018.1549100](https://doi.org/10.1080/00031305.2018.1549100).
